# Supplementary material for: Inter-dependent apical microtubule and actin dynamics orchestrate centrosome retention and neuronal delamination
Source: eLife. 2017 Oct 23;6:e26215. doi: 10.7554/eLife.26215 (PMC5653239; doi:10.7554/eLife.26215)
Supplement: Transparent reporting form [file elife-26215-transrepform.docx]

***eLife’s* transparent reporting form**

We encourage authors to provide detailed information *within their submission* to facilitate the interpretation and replication of experiments. If you have any questions, please contact us: [editorial@elifesciences.org](mailto:editorial@elifesciences.org).

**Sample-size estimation**

- You should state whether an appropriate sample size was computed when the study was being designed
- You should state the statistical method of sample size computation and any required assumptions
- If no explicit power analysis was used, you should describe how you decided what sample (replicate) size (number) to use

Please outline where this information can be found within the submission (e.g., page numbers or figure legends), or explain why this information doesn’t apply to your submission:

Please see the sections below.

**Replicates**

- You should report how often each experiment was performed
- You should include a definition of biological versus technical replication
- The data obtained should be provided and sufficient information should be provided to indicate the number of independent biological and/or technical replicates
- If you encountered any outliers, you should describe how these were handled
- Criteria for exclusion/inclusion of data should be clearly stated
- High-throughput sequence data should be uploaded before submission, with a private link for reviewers provided (these are available from both GEO and ArrayExpress)

Please outline where this information can be found within the submission (e.g., page numbers or figure legends), or explain why this information doesn’t apply to your submission:

Update for revised manuscript in bold

- The biological replicate we have assessed here is the behaviour of or structures in individual cells. We present our data in terms of numbers of cells, observed in numbers of explants/slices, which were taken from numbers of embryos. In all cases only one neural tube explant is taken from each chick embryo, while multiple (2 or 3) embryo slices may be taken from individual embryos. All experiments in which tissues were treated with small molecules were carried out multiple times on different days and these are considered as independent experiments. We do not generate technical replicates with the live imaging assays presented in this paper.
- The information relating to number of independent experiments, cells, explants and embryos sampled is listed below in the order in which these data are presented in the paper. Numbers of cells, explants and embryos are found in the text of the Results (while number of experiments is given in the figure legends), except for quantifications for Nocodazole and Latrunculin-A experiments, all data for which are presented in Figure 3 legend.
- Measurement of microtubule wheel diameter: 21 cells were measured in 2 explants from 2 embryos (page 7).
- Apical microtubule architecture in mouse embryo spinal cord or cortex, 32 fields of view each consisting of ~30 cells, were imaged in 2 explants of each tissue from 2 embryos (page 7)
- En face imaging of apical microtubule architecture in chick embryos: 167 cells were imaged in 4 explants from 4 embryos (page 7)
- **Actin-tubulin alignment across the adherens junctions at the N-Cadherin level: A line was drawn across two cells. Thirty-one such measurements were performed for a total of 3 explants from 3 embryos (3 experiments) (page 7)**
- Structured illumination microscopy of the EMTB labelled apical microtubules was carried out on 4 cells from 2 embryos (page 8)
- EB3-GFP dynamics was monitored in 2 independent experiments. The first with PACT-TagRFP (51 cells imaged in 3 explants from 3 embryos) and the second, with F-tractin-mKate2 (95 cells imaged in 3 explants from 3 embryos) (pages 8 and 9).
- **Quantification of EB3-GFP / F-tractin-mKate2 inter-peak distance: 10 cells from 3 explants from 3 embryos (1 experiment) (page 9)**
- Effects of Nocodazole or Latrunculin-A at the adhesion belt, microtubules and apical endfoot size were assessed in 3 independent experiments (with the exception of DMSO control for Latrunculin-A where measurements in 2 experiments were used). All measurement and explant numbers for these experiments are provided in detail in figure legend 3 (pages 9 and 10).
- EB3-GFP dynamics in cells with small apical end foot were assessed in 6 cells in 4 explants from 4 embryos) (page 10)
- STED imaging of EMTB-GFP and F-tractin-mKate2 composite ring was carried out on 4 cells from 2 embryos (page 10 and 11)
- Microtubule dynamics during delamination was assessed in 8 independent experiments (22 cells, 15 slices) (page11)
- Effects of DMSO control or the small molecules (Nocodazole and Taxol) on delamination was assessed in at least 5 independent experiments (with slices taken from 2 or 3 embryos for each experiment). DMSO control for Nocodazole experiments: 61 cells in 29 slices; Nocodazole: 51 cells in 35 slices; DMSO control for Taxol experiments: 55 cells in 23 slices; Taxol: 51 cells in 26 slices) (pages 11 and 12).
- **Effects of Stathmin-GFP or EGFP control on delamination was assessed as follows: For Stathmin-GFP 11 cells in 6 slices, 5 experiments and for the EGFP control 17 cells in 9 slices, 6 experiments (page 12).**
- Microtubule and actin dynamics during delamination was assessed in: 12 cells, 6 slices, 5 embryos (page 13).
- Quantification of EMTB-GFP or F-tractin-mKate2 dynamics for each experimental condition was assessed in least 5 independent experiments. EMTB-GFP in DMSO control for Taxol (12 cells in 11 slices) and Taxol (12 cells in 10 slices). F-tractin-mKate2 in DMSO control for Taxol (12 cells in 11 slices) and Taxol (20 cells in 16 slices). EMTB-GFP in water control for ML-7 (10 cells in 10 slices) and ML-7 (16 cells in 13 slices) (pages 13 and 14).
- Cells exhibiting sub apical constrictions in taxol treated slices were assessed in 8 independent experiments (34 cells in 21 slices) (pages 13 and 14).
- Cells exhibiting sub apical constrictions in ML-7 treated slices were assessed in 9 independent experiments (25 cells in 18 slices) (page 14).
- **Endogenous Drebrin localization in transverse sections was assessed in 3 sections from each of 4 embryos (page 15).**
- **Localization of Drebrin in apical end-feet of cells electroporated with Drebrin-mCherry and EMTB-GFP and stained for actin: 18 cells in 6 embryos (page 15)**
- **Co-localisation with the actin belt and the apical EMTB-GFP-labelled microtubules was quantified in 7 cells from 3 embryos (page 15)**
- **En face imaging of Drebrin-YFP or Drebrin-mcherry, was assessed in one experiment for each construct and a total of 5 explants from 5 embryos (page 15).**
- **Effects of Drebrin-shRNA or scrambled control on delamination was assessed as follows: For Drebrin-shRNA 27 cells in 9 slices, 5 experiments and for the scrambled control 10 cells in 7 slices, 4 experiments (page 15).**
- Centrosome moving through constricted sub-apical membrane were assessed in 10 cells, 9 slices, 9 embryos (page 16).
- Microtubule and centrosomal dynamics in delaminating cells was assessed in 4 independent experiments (8 cells in 8 slices) (page 16)
- Average centrosome diameter measured with IFT88 (36 cells in 2 explants from 2 embryos (page 16)
- Measurement of the condensed microtubule ring visualised with acetylated alpha tubulin (17 cells in 2 explants from 2 embryos) page 16
- Centrosomal localization in DMSO control, Taxol or ML-7 was assessed in at least 3 independent experiments (DMSO control for Taxol experiments 26 cells in 14 slices; Taxol: 24 cells in 12 slices, ML-7: 31 cells in 9 slices (page 17).
- KillerRed experiments: Demonstration of centrosome disruption (loss of PACT-YFP): 2 experiment, 5 cells, 5 slices, 5 embryos. Control replacing PACT-Killer-Red with PACT-TagRFP: 1 experiment, 25 cells, 4 slices, 4 embryos (pages 17 and 18),
- Microtubule nucleation (EB3-GFP) following CALI was assessed in 3 independent experiments: 22 cells in 7 explants from 7 embryos (page 18).
- Effect of PACT-KillerRed/CALI and control PACT-TagRFP: CALI on delamination assessed in 5 independent experiments: 12 cells, 5 slices, 5 embryos; 25 cells, 10 slices (page 18).

**Statistical reporting**

- Statistical analysis methods should be described and justified
- Raw data should be presented in figures whenever informative to do so (typically when N per group is less than 10)
- For each experiment, you should identify the statistical tests used, exact values of N, definitions of center, methods of multiple test correction, and dispersion and precision measures (e.g., mean, median, SD, SEM, confidence intervals; and, for the major substantive results, a measure of effect size (e.g., Pearson's r, Cohen's d)
- Report exact p-values wherever possible alongside the summary statistics and 95% confidence intervals. These should be reported for all key questions and not only when the p-value is less than 0.05.

The statistical analysis section in the Materials and Methods is on page 33:

For Figure 2, the paired t-test was used to compare the inter-peak distance between EB3-GFP comets and F-tractin-mKate2 for the different timepoints (page 45)

For Figure 3, the t-test was used for the comparison of the mean area under the curve, apical endfoot area and the tubulin fluorescence (pages 46-47).

For Figure 6, the 2-way ANOVA test was used for the comparison of the curves’ trend when EMTB-GFP and F-tractin-mKate2 were monitored following treatment with small molecules or their controls and fluorescence was then quantified over time (pages 51-52).

Please outline where this information can be found within the submission (e.g., page numbers or figure legends), or explain why this information doesn’t apply to your submission:

(For large datasets, or papers with a very large number of statistical tests, you may upload a single table file with tests, Ns, etc., with reference to page numbers in the manuscript.)

**Additional data files (“source data”)**

- We encourage you to upload relevant additional data files, such as numerical data that are represented as a graph in a figure, or as a summary table
- Where provided, these should be in the most useful format, and they can be uploaded as “Source data” files linked to a main figure or table
- Include model definition files including the full list of parameters used
- Include code used for data analysis (e.g., R, MatLab)
- Avoid stating that data files are “available upon request”

Please indicate the figures or tables for which source data files have been provided:

N/A
